# The Effects of Combined Motor Control and Isolated Extensor Strengthening Versus General Exercise on Paraspinal Muscle Morphology, Composition, and Function in Patients with Chronic Low Back Pain: A Randomized Controlled Trial

**DOI:** 10.3390/jcm12185920

**Published:** 2023-09-12

**Authors:** Maryse Fortin, Meaghan Rye, Alexa Roussac, Chanelle Montpetit, Jessica Burdick, Neda Naghdi, Brent Rosenstein, Cleo Bertrand, Luciana G. Macedo, James M. Elliott, Geoffrey Dover, Richard DeMont, Michael H. Weber, Véronique Pepin

**Affiliations:** 1 Department of Health, Kinesiology and Applied Physiology, Concordia University, Montreal, QC H4B 1R6, Canada; meaghanrye@gmail.com (M.R.); alexarou@live.ca (A.R.); montpetitchanelle@gmail.com (C.M.); jessicaburdick@hotmail.com (J.B.); nd.naghdi@gmail.com (N.N.); brent.rosenstein@icloud.com (B.R.); cleobertrand01@gmail.com (C.B.); geoffrey.dover@concordia.ca (G.D.); richard.demont@concordia.ca (R.D.); veronique.pepin@concordia.ca (V.P.); 2 School of Health, Concordia University, Montreal, QC H4B 1R6, Canada; 3 School of Rehabilitation Science, Faculty of Health Sciences, McMaster University, Hamilton, ON L8S 1C7, Canada; macedol@mcmasters.ca; 4 Faculty of Medicine and Health, School of Health Sciences, The Kolling Institute, University of Sydney, Sydney, NSW 2050, Australia; jim.elliott@sydney.edu.au; 5 Northern Sydney Local Health District, St. Leonards, NSW 2065, Australia; 6 Department of Orthopedic Surgery, McGill University Health Centre, Montreal, QC H3J 1A4, Canada; michael.weber@hotmail.com

**Keywords:** low back pain, motor control, paraspinal muscles, imaging, magnetic resonance imaging, ultrasound, fatty infiltration, composition, function

## Abstract

Low back pain (LBP), a globally widespread and persistent musculoskeletal disorder, benefits from exercise therapy. However, it remains unclear which type leads to greater changes in paraspinal muscle health. This study aimed to (1) compare the effects of a combined motor control and isolated lumbar extension exercise (MC+ILEX) versus a general exercise (GE) intervention on paraspinal muscle morphology, composition, and function, and (2) examine whether alterations in paraspinal muscle health were correlated with improvements in pain, function, and quality of life. Fifty participants with chronic LBP were randomly assigned to each group and underwent a 12-week supervised intervention program. Magnetic resonance imaging and ultrasound assessments were acquired at baseline, 6 and 12 weeks to examine the impact of each intervention on erector spinae (ES) and multifidus (MF) muscle size (cross-sectional area, CSA), composition, and function at L4-L5 and L5-S1. Self-reported questionnaires were also acquired to assess participant-oriented outcomes. Our findings indicated that the MC+ILEX group demonstrated greater improvements in MF and ES CSA, along with MF thickness at both levels (all *p* < 0.01). Both groups significantly improved in pain, function, and quality of life. This study provided preliminary results suggesting that an MC+ILEX intervention may improve paraspinal morphology while decreasing pain and disability.

## 1. Introduction

Low back pain (LBP) is the main contributor to disability globally [1]. Over 80% of the population worldwide will experience LBP at some point in their lives, which has a significant economic impact on individuals, healthcare systems, and local economies [2,3,4,5]. While the multifactorial biopsychosocial–environmental etiology is widely recognized [6,7,8], there remain limited options for effective conservative management programs despite nearly 30 years of data from clinical trials [9]. Imaging studies demonstrate that people with LBP are more likely to present with morphological and compositional asymmetries, including muscle fatty infiltration and paraspinal muscular atrophy, especially in the multifidus muscle as compared to healthy controls [10,11,12]. Evidence suggests that people with chronic LBP have decreased multifidus muscle cross-sectional areas (CSA, morphology) and increased fatty infiltration (composition) at a single or multiple levels of the lumbar spine [13,14]. In tandem with these muscular changes, a decrease in muscle strength, endurance, and activation patterns in the hip and back musculature has been observed. However, causal inferences between the multifidus morphological and functional changes are unclear [15,16,17].

A significant percentage of people with chronic LBP seek physical therapy care to alleviate their pain and improve their functional capacity [17,18]. Physical therapy plays a central and pivotal role within an integrated multidisciplinary treatment strategy for chronic LBP, synergizing with instrumental therapies, medications, and injections, as highlighted in previous studies [19,20,21]. The cohesive approach of LBP management highlights the significance of physical therapy interventions in addressing both the physical and psychosocial dimensions of the condition. Exercise therapy is recommended as an initial approach for individuals experiencing chronic LBP, with evidence for improved pain, quality of life, depression, and disability status [17,22,23,24,25,26,27,28]. Given the body of evidence linking LBP to muscular changes (e.g., atrophy, fatty infiltration, asymmetry) in the trunk and paraspinal muscles, many exercise therapies focus on improving activation and control of these muscles [11,22,25,26,29,30]. In a recent study, the effects of a high-intensity intervention centered on isolated lumbar extensor exercise examined muscle cross-sectional area (CSA) and fatty infiltration of the multifidus and erector spinae muscles [31]. Variability in both morphological and compositional changes was found, where some individuals experienced increased CSA and reduced fatty infiltration, while others exhibited no alterations [31]. Another study found that a 16-week progressive, free-weight exercise intervention significantly reduced lumbar paraspinal muscle fat infiltration [32]. On the other hand, a systematic review examined the impacts of motor control exercise on the structural characteristics of the lumbar multifidus muscle and LBP and found preliminary evidence to support that motor control exercises may positively affect multifidus size, with a positive dose–response relationship [33]. Currently, there is a scarcity of studies that have comprehensively investigated the effects of exercise therapy interventions on the overall paraspinal muscle health (morphology, composition, and function), and how these changes may be linked with improvements in pain and disability [1].

Whilst there is moderate quality evidence indicating that exercise therapy leads to greater reduction in pain and improvement in function compared to control or minimal intervention [34,35], studies comparing the effects of exercise interventions on paraspinal muscle morphological and compositional changes and its association with pain and disability are scarce [1]. Indeed, while motor control exercise and resistance training are among the most popular and promising forms of exercises to improve paraspinal muscle quality and restore paraspinal muscle activation/motor control, most exercise trials only report on changes in patient-oriented outcomes. To the best of our knowledge, Berry et al. is the only study that examined the effects of a high-intensity resistance intervention on the lumbar muscles in individuals with LBP and reported a correlation between MRI changes and patient-related outcomes [31]. Individuals who presented with enhancements in muscle health also showed the greatest improvements in function, muscle strength, depression, and anxiety [31]. While there is a widely held belief that improving paraspinal muscle quality leads to better patient-related outcomes, this theory has not been thoroughly assessed and warrants further attention.

The objectives of this study were to (1) compare the effects of a combined motor control and isolated lumbar extension strengthening group (MC+ILEX) versus a general exercise group (GE) on (i) paraspinal (multifidus and erector spinae) muscle morphology and composition (size, fatty infiltration) and (ii) overall multifidus muscle function (% thickness change from at rest to a contracted position) and (2) investigate the association between the observed changes in muscle morphology with the changes in pain and disability postintervention. We hypothesized that participants in the MC+ILEX group would show significant improvements in the overall health of paraspinal muscle health (multifidus muscle morphology, composition, and function). We also hypothesized that the positive changes in paraspinal muscle health would be associated with concomitant improvements in patient-related outcomes (pain and disability).

## 2. Materials and Methods

### 2.1. Study Design and Setting

**Figure A1:**
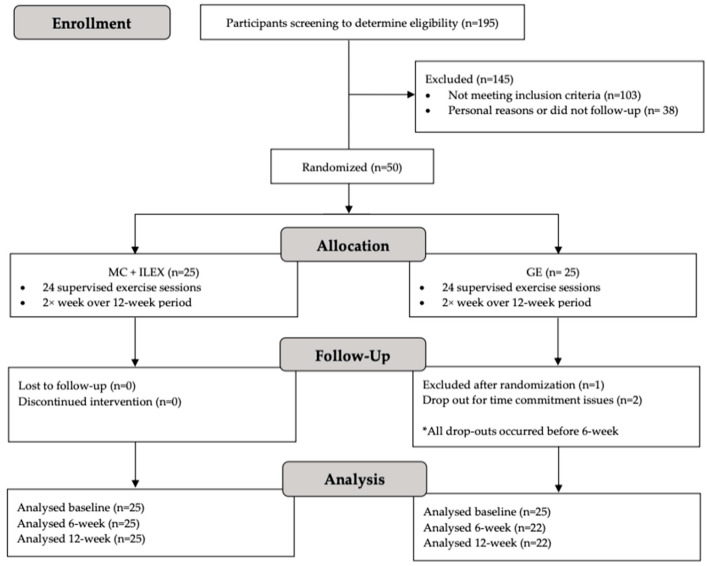
Consort flow diagram.

This study was a two-arm randomized control trial (RCT) with a test–retest design (refer to Figure A1 in the Appendix A). The study protocol has been previously published [1], and the trial was prospectively registered (NTCT04257253). This monocentric study was conducted at Concordia University’s School of Health and was approved by the Central Ethics Research Committee under the jurisdiction of the Quebec Minister of Health and Social Services (#CCER-19-20-09). Each participant provided their informed consent by signing a consent form. The study was reported following the CONSORT statement [36].

### 2.2. Participants

Individuals were eligible for enrollment in this study if they satisfied all the following criteria for inclusion: (1) nonspecific chronic low back pain (LBP) lasting at least 3 months (accompanied with or without leg pain), (2) between the ages of 18 and 65 years old, (3) communicated in either the English or French language, (4) were in the pursuit of care for LBP, (5) achieved a ranking of “moderate” or “severe” on the modified Oswestry Low Back Pain Disability Questionnaire, (6) had not participated in any sport or training targeting the muscles of the lower back within the 3 months preceding the start of the trial. Individuals were excluded if they fulfilled any of the following criteria: (1) any indications of nerve root compression or observable deficits in motor reflexes; (2) prior spinal surgery, lumbar steroid injections, or fractures of the vertebrae; (3) significant structural irregularities in the lumbar spine (e.g., spondylosis, spondylolisthesis, scoliosis > 10°); (4) pregnancy; (5) medical conditions that hinder the safe participation in physical exercise as evaluated by the Physical Activity Readiness Questionnaire. 

### 2.3. Participant Recruitment

Participants were enlisted from the nearby university community through email advertising and from the Quebec LBP Consortium, a group of experts from diverse disciplines aiming to establish a province-wide online database with longitudinal data of individuals with LBP [37]. Participants who expressed interest in the study were contacted by a member of the research team to confirm eligibility and enroll the participants. Participant recruitment started in October 2020 and the data collection was completed by October 2021.

### 2.4. Randomization and Blinding

Participants were assigned randomly to either treatment group in a balanced manner (1:1). The random allocation was achieved through consecutively numbered opaque sealed envelopes (generated by a computerized randomization sequence with permuted blocks) prepared by an individual not involved in the study. Solely the assessor was blinded to participants’ characteristics for imaging, as concealing this information from both the therapists and participants is typically unfeasible in exercise intervention studies [38]. 

### 2.5. Procedure

Participants in both groups underwent a 12-week intervention program involving two supervised exercise sessions weekly, each lasting approximately 45 min. The interventions were delivered by a certified athletic therapist (MC+ILEX) and a graduate student in exercise science (GE) with 1 year of experience. Participants in both groups were advised to follow a home exercise program during the intervention and after discharge. The home program was different for each group and included exercises similar to the main intervention but performed with elastic bands. Participants in both groups were instructed to perform the home program 2–3×/week. Throughout the intervention period, participants were asked to avoid seeking other forms of treatment (e.g., chiropractor, osteopath, massage) and medication, although this did not hinder participation. Participants were asked to report any cointerventions at the end of the trial.

### 2.6. Intervention Protocols

#### 2.6.1. General Exercise Group (GE)

Participants in the GE group underwent a 12-week program that started with a 10 min aerobic warmup (stationary bike or incline treadmill walking), followed by resistance training exercises for the glutes, hip adductors/abductors, and upper back muscles (e.g., rhomboids, latissimus dorsi) and finally trunk-leg stretches. The machine-based resistance training program was structured as a two-day split routine, concentrating on distinct muscle groups for each session (refer to Table A1 in the Appendix A). The intensity of the intervention systematically increased throughout its duration based on a study methodology established by Iversen et al. [39]. The designated repetitions were as outlined: weeks 1–2, 15–20 repetitions; weeks 3–5, 12–15 repetitions; week 6–8, 10–12 repetitions; weeks 9–12, 8–10 repetitions. Each exercise was performed for three sets and the weights were increased by 5% when participants managed to accomplish 2 or more repetitions than the designated range. At the end of each session, stretches such as cat–cow, pigeon, deep lunge, and piriformis were performed. In accordance with the American College of Sports Medicine (ACSM) guidelines, all stretch positions were held for a duration of 10 s and repeated 3 times per side [40]. The aim of this intervention was to facilitate participants in resuming their regular activities of daily living, which encompass tasks like standing, lifting, and walking. Evidence suggests that general exercise programs emphasizing lower-body strength and flexibility are effective in producing these outcomes [24].

#### 2.6.2. Combined Motor Control and Isolated Lumbar Extension Group (MC+ILEX)

The aim of the intervention was to restore proper control, coordination, and synergistic contraction of the lumbar muscles, with the goal of enhancing spinal stability both while at rest and during various movements [41,42]. The intervention was based on motor control principles, starting with the cognitive phase (initiating activation of the deep spinal muscles) and transitioning to the associative and autonomous phase ultimately progressing towards functional rehabilitation [43,44]. In addition, the intervention also incorporated coordination and optimal control of global trunk muscles [45,46]. 

#### 2.6.3. Phase 1: Cognitive Phase

The initial phase of the intervention began by evaluating muscle engagement and breathing patterns. Afterward, a motor control regimen was implemented to address any deficits identified in the assessment. The main aim of the intervention was to correct certain muscle patterns such as increasing the deep trunk muscle activation (multifidus and transverse abdominus muscles) while reducing the engagement of global muscles (refer to Table A2 in the Appendix A) [42,45,46]. Deep trunk muscle activation was accomplished through a series of increasingly difficult positions. As a prerequisite to progress to stage two, participants were required to fulfill the following conditions: perform 10 repetitions while holding for 10 s, attain activation with minimal guidance or prompts, and maintain regular breathing patterns during the exercises [47]. Both phases of the intervention included the correction of breathing patterns with an emphasis on diaphragmatic breathing. 

#### 2.6.4. Phase 2: Autonomous Activation Phase

Once the participants effectively activated the deep trunk muscles with minimal superficial muscle compensation, while maintaining proper breathing, they progressed to phase 2. This phase focused on gradually increasing the load placed on the muscles, from static to dynamic positions, while maintaining a neutral alignment of the lumbar region and ensuring coordination of the deep core muscles [46]. Progression in exercise was accomplished by placing the participant in increasingly demanding positions (supine to sitting), intensifying the resistance (limb movement), and introducing elements of dynamic stability (unstable surface). The aim of this phase was to achieve automatic engagement of deep trunk muscles while fostering synergistic coordination with the superficial muscles. 

**Figure A2:**
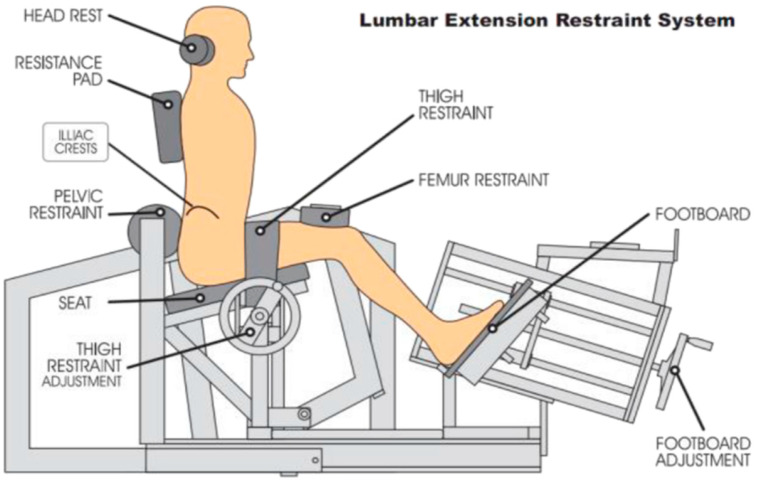
Diagrammatic representation of the MedX Lumbar medical machine. Image reprinted with permission [48].

Participants within this group also completed isolated lumbar extensor strength exercises (ILEX) in conjunction with the motor control exercises. The resisted training session was completed on the MedX machine (refer to Figure A2 in the Appendix A) [48]. The baseline testing involved measuring the participants’ one repetition maximum (1 RM). During the intervention, participants completed two sets of lumbar extension with 15–20 repetitions at 55% of their 1 repetition maximum (1 RM) at a 24° angle. When the participant successfully completed 15–20 repetitions, the load was augmented by 5% [49,50]. The MedX lumbar extensor machine was designed to enable testing and strengthening of the lumbar extensor muscles in the flexion–extension plane of movement over the entire range. By providing pelvic and lower body stabilization, the need for the engagement of compensatory and synergistic muscles (e.g., glutes and hamstrings) was eliminated, thereby allowing for isolated lumbar extensor strengthening.

### 2.7. Outcome Measures

All outcome variables were collected at baseline, 6 weeks, and 12 weeks for participants in both intervention groups. All self-reported questionnaires were completed on paper in person. MRI and ultrasound assessments of the lumbar extensor muscle assessments were obtained at each time point at Concordia University’s School of Health. Demographic characteristics were collected at baseline. 

### 2.8. Primary Outcome

#### Multifidus Muscle Morphology

Multifidus muscle morphology (size and fatty infiltration) was examined at L4-L5 and L5-S1 using IDEAL (lava-flex, 2-echo) fat–water sequences acquired via a 3-Tesla General Electric (Chicago, IL, USA) MRI machine (standard phase array body coil with 4 mm slice thickness, 512 × 512 matrix and 180 mm^2^ field of view).

The multifidus cross-sectional area (CSA) was traced manually on corresponding fat and water images to assess muscle size. The percent fat signal fraction was calculated from the CSA segmentation using the following formula: %FSF = (Signal_fat_/[Signal_water_ + Signal_fat_] × 100). Measurements were obtained on 3 slices per level (upper endplate, mid-disc, and lower endplate). The mean of the CSA and %FSF for each level (L4-L5 and L5-S1) was used in the analyses. Intrarater correlation coefficients (ICC_1,3_) were calculated using a sample of 10 images, a minimum of 5 days apart, with excellent reliability (ICC: 0.96–0.99) for both CSA and %FSF at both spinal levels.

### 2.9. Secondary Outcomes 

#### 2.9.1. Multifidus Muscle Function 

Muscle thickness was assessed using an Aixplorer Supersonic ultrasound machine with a curvilinear 1–6 MHz transducer. The thickness of the multifidus muscle was analyzed in both its resting state and during submaximal contraction, involving measurements taken during contralateral arm lifts at the L4-L5 and L5-S1 levels. To evaluate submaximal contraction, the participant was guided to lift their arm while grasping a handheld weight (adjusted according to the participant’s body weight) in a prone position whilst the evaluator used ultrasound to examine the contralateral multifidus [51,52]. The following equation was used to measure the change in multifidus muscle thickness from submaximal (rest) to maximal (contracted) states = (thickness_contracted_ − thickness_rest_)/thickness_rest_) × 100. This method for evaluating the multifidus using ultrasound is reliable and valid [51,53,54]. Measurements were repeated three times on each side and the mean was used for the analysis. Intrarater correlation coefficients (ICC_1,3_) were calculated using a sample of 10 images, a minimum of 5 days apart, with excellent reliability (ICCs: 0.90–0.99) for both thickness and %thickness changes at both spinal levels. 

#### 2.9.2. Erector Spinae Muscle Morphology

The erector spinae muscle CSA at L4-L5 and L5-S1 was traced manually on corresponding fat and water images to assess muscle size, as described above for the multifidus muscle, and the reliability was comparable to the multifidus muscle. 

#### 2.9.3. Disability

The Oswestry Disability Index (ODI) was used to evaluate participants’ self-reported levels of disability associated with LBP and disability. Each item on this 10-item scale required a rating from 0 to 5. A score of 0 indicated that the pain had no impact the situation, while a score of 5 indicated severe disability. The questionnaire covers categories such as pain intensity, personal care, lifting, walking, sitting, standing, sleeping, sex life, social life and traveling. Scores were classified into different groups, including minimal, moderate, severe, disabled, or bedridden. The ODI is a core set outcome for measuring low back pain-related disability due to its reliability and validity [55,56]. 

#### 2.9.4. Health-Related Quality of Life

The participants’ health-related quality of life was assessed using the 12-item Short Form Health Survey (SF-12). The 12-item survey uses eight domains to asses various dimensions of health, encompassing both physical and mental aspects: (1) restrictions in physical activities due to health concerns, (2) restrictions in social engagements due to physical or emotional concerns, (3) restrictions in usual role-related activities due to physical health concerns, (4) bodily pain, (5) overall mental health (encompassing psychological stress and well-being), (6) restrictions in usual role-related activities due emotional concerns, (7) vitality (comprising energy levels and fatigue), and (8) general perceptions of health. The scores from each question were summed to obtain an overall score ranging from 0 to 100, with 100 being the best mark of health. The SF-12 is both a reliable and valid tool [57,58].

#### 2.9.5. Pain

The Numerical Pain Rating Scale (NPR) was used to evaluate the degree of pain experienced by participants on a scale from 0 to 10. The scale is a reliable and valid method of detecting changes in perceived pain [59,60]. 

#### 2.9.6. Adherence

The therapists assessed the adherence to each intervention by recording in the treatment files the number of attended sessions (out of a maximum of 24 sessions) for each participant. Adherence to the home exercise program was assessed and noted by the therapists at the end of the intervention by asking the following question “How often did you perform your home exercises” and using an ordinal scale: (1) none of the time, (2) some of the time, (3) most of the time, (4) almost all of the time, (5) all of the time.

### 2.10. Statistical Analysis

An a priori sample size calculation was established based on the effect size (significant pre–post-difference in CSA measurements of the multifidus muscle following a motor control intervention) obtained from a prior study [61]. G*power software (version 3.1) was utilized to calculate the sample size based on a mean effect size of d = 0.90, a power of 80%, a significance level of alpha 0.05, and incorporating a 10% buffer for potential loss to follow-up and 10% treatment nonadherence. Baseline characteristics were evaluated using descriptive statistics. Changes multifidus and erector spinae size, composition, and function from pre- to postintervention were analyzed using a between-subjects repeated measures ANOVA, while adjusting for baseline values. Repeated measures ANOVA was also used to assess the changes in participant-oriented outcomes. Pearson’s correlations were used to analyze the correlation between changes in multifidus muscle and erector spine CSA and %FSF with changes in related disability and pain postintervention. Interpretation of the correlation strength was based on Cohen’s conventions, categorizing correlations as small, moderate, and strong when they were approximately 0.10, 0.30, and 0.50, respectively [62]. All statistical analyses were completed using IBM SPSS (version 28.0.0.0 (190), New York, NY, USA); a *p*-value of <0.05 was considered statistically significant.

## 3. Results

### 3.1. Demographics and Adherence

One hundred ninety-five individuals expressed their interest to take part in the trial. Thirty-eight declined to participate in the study due to personal reasons or failed to maintain contact to be further screened. One hundred and three individuals were ineligible due to not meeting the specified inclusion criteria. A flowchart diagram is presented in Figure A1 (Refer to Appendix A). The most frequent reason for exclusion was a low-to-moderate score on the ODI, followed by having a preexisting spinal abnormality. Initially, four participants were enrolled but later, excluded due to the identification of spinal abnormalities on the baseline MRI. All participants that were randomly allocated were included in the primary (intention to treat) analysis independent of compliance and loss to follow-up.

In total, 50 participants were recruited and randomly assigned to each group (*n* = 25 in MC-ILEX, *n* = 25 in GE). Each of the 25 participants allocated to the MC+ILEX intervention successfully completed the 12 weeks intervention (no dropout). Among the initial group of 25 participants allocated to the GE intervention, a total of 22 participants completed the 12-week intervention. One participant was excluded from the study after randomization and two dropped out due to conflicting time commitments, all within 2 weeks of starting the trial. The MC+ILEX group reported a mean attendance of 22.1 ± 1.6 and the GE group reported a mean attendance of 22.3 ± 1.3 out of a possible 24 sessions, indicating a high level of participation in this study. With regards to the home exercise program, most participants in the MC+ILEX group reported doing the home exercise program either “some of the time” (44%), “most of the time” (16%), or “almost all the time” (16%). Similarly, most participants in the GE group reported performing the home exercise program either “some of the time” (29%) or “most of the time” (33%). Baseline demographic characteristics between groups appeared similar (Table 1). Both groups displayed large variability in the self-reported LBP duration, with the mean duration of LBP reported as 73.5 ± 82.8 months in the MC+ILEX group and 101.7 ± 105.6 months in the GE group.

### 3.2. Effect of MC+ILEX and GE on Muscle Cross-Sectional Area (CSA)

**Figure 1:**
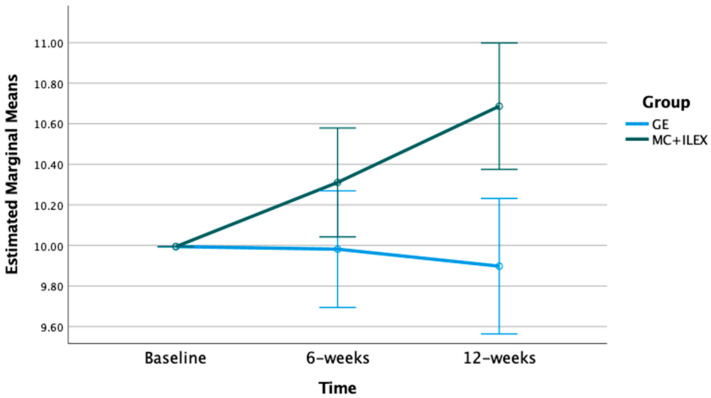
Bar graph of the mean changes in multifidus CSA at the L4/L5 level in both groups, with standard error, adjusted for baseline values.

**Figure 2:**
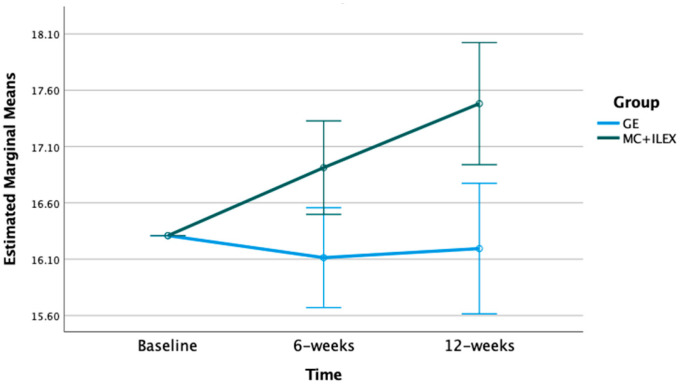
Bar graph of the mean changes in erector spinae CSA at the L4/L5 in both groups, with standard error, adjusted for baseline values.

**Figure 3:**
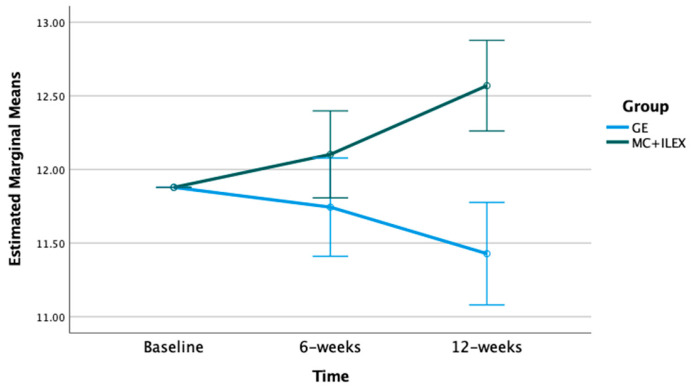
Bar graph of the mean changes in multifidus CSA at the L5/S1 levels in both groups, with standard error, adjusted for baseline values.

**Figure 4:**
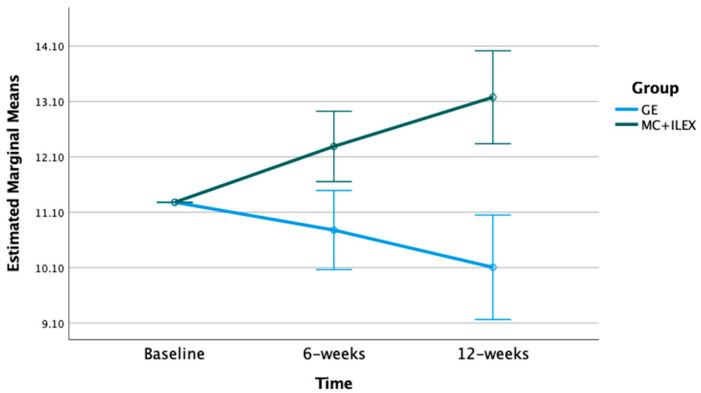
Bar graph of the mean changes in erector spinae CSA at L5/S1 in both groups, with standard error, adjusted for baseline values.

The mixed model ANOVA with repeated measures indicated significant time*group interactions for the multifidus and erector spinae CSA at the L4-L5 (*p* < 0.01) and L5-S1 levels (*p* < 0.001) (refer to Figure 1, Figure 2, Figure 3 and Figure 4). Postintervention, participants in the MC+ ILEX group had a greater increase in the CSA of the multifidus (mean difference [95% CI]) at L4/L5 (0.69 [0.38–1.00] cm^2^), erector spinae at L4-L5 (1.17 [0.63–1.71] cm^2^), multifidus at L5/S1 (0.69 [0.39–1.00] cm^2^), and erector spinae at L5/S1 (1.90 [1.06–2.73] cm^2^) compared to the GE group (Table 2).

### 3.3. Effect of MC+ILEX and GE on Fatty Infiltration (% Fat Fraction)

The repeated measures ANOVA indicated no significant time*group interactions for the erector spinae and multifidus %FF at both the L4-L5 and L5-S1 levels (all *p* > 0.05). No significant change in %FF of the multifidus muscle at L4/L5 and L5/S1 (Table 3) was observed throughout the intervention for either group. Technical imaging issues prevented the reconstruction of five sets of IDEAL images, leading to the impossibility of calculating fat fraction, and limiting the sample size to 45 for this measure.

### 3.4. Effect of MC+ILEX and GE on Multifidus Thickness and Function

The repeated measures ANOVA revealed significant time*group interactions for the multifidus thickness at L4 (*p* < 0.001) and L5 levels (*p* < 0.001). Postintervention, participants in the MC+ ILEX group had greater multifidus thickness (mean difference [95% CI]) at the L4 (0.22 [0.15–0.29] cm^2^) and L5 (0.25 [0.18–0.32] cm^2^) levels compared to the GE group (Table 4). In the MC+ILEX group, multifidus thickness showed a significant increase at both L4 and L5 across all time points. However, the GE group displayed no changes at either L4 or L5 levels. No significant differences were observed in the multifidus muscle thickness % changes between resting and contracted states in either group (Table 5).

### 3.5. Effect of MC+ILEX and GE on Self-Reported Outcomes

The repeated measures ANOVA revealed no significant time*group interactions for the NPR scores (*p* = 0.34), ODI scores (*p* = 0.84), SF-12 physical health scores (*p* = 0.32), and SF-12 mental health scores (*p* = 0.37) (Table 6). There were, however, significant improvements in self-reported pain levels (NPR, *p* < 0.001) and function (ODI, *p* < 0.001) across all timepoints in the MC+ILEX group. Significant improvements between baseline and 6 weeks and baseline and 12 weeks in self-reported pain levels (NPR, *p* < 0.001) and across all time points for function (ODI, *p* < 0.001) were also observed in the GE group. Notably, the MC+ILEX group demonstrated significant improvements in physical health (SF-12 PCS, *p* = 0.004) from baseline to both the 12-week mark and from 6 weeks to 12 weeks. Significant improvements in physical health (SF-12 PCS, *p* = 0.025) were noted in the GE group, specifically between baseline to 6 weeks and baseline to 12 weeks. Likewise, the GE group experienced significant improvements in mental health (SF-12 MCS, *p* = 0.074) from baseline to 12 weeks.

### 3.6. Correlation between Muscle Morphology and Clinical Outcomes

Significant moderate correlations were present between L4 multifidus thickness and SF-12 mental health scores (r = −0.31, *p* = 0.04) as well as L5 multifidus thickness and SF-12 mental health scores (r = −0.37, *p* = 0.01). Table 7 displays the Pearson correlations between changes in muscle morphology and changes in self-reported measures from baseline to 12 weeks.

## 4. Discussion

Our findings revealed a significant between-group difference for multifidus and erector spinae CSA, with a significant increase in CSA only observed in the MC+ILEX group from baseline to postintervention at the L4/L5 and L5/S1 levels. Contrary to the inconsistent results observed in previous studies regarding the effects of exercise interventions on the size of the paraspinal muscles among individuals with LBP, we observed a consistent increase in the multifidus and erector spinae muscle CSA in the MC+ILEX group. The consistent effect could potentially be attributed to the unique characteristics of the intervention. The mean change for multifidus and erector spinae CSA did reach the minimum detectable change for both levels (e.g., MDC multifidus = 0.49–0.60; MDC erector spinae = 0.39–1.9) [63] suggesting that the observed changes for both muscles were beyond measurement error.

In accordance with our findings, Shahtahmassebi et al. evaluated the effects of trunk strengthening and motor control exercises in a multimodal exercise program and found an increase in lumbar multifidus muscle size following the 12-week program [64]. However, the participants included in the latter study were overall healthy and moderately active older adults, which makes it harder to generalize to other populations. Conversely, Berry et al. examined the effect of a 10-week ILEX intervention with the MedX machine (20 sessions, one set of 15–20 repetitions at 60–80% of maximum effort) and reported no change in the multifidus or erector spinae CSA postintervention, despite improvements in strength and reduced pain [31]. The varying results could be attributable to the inclusion of motor control training for the trunk muscles of our intervention. Indeed, motor control was established within a mechanical framework of LBP and is based on substantial evidence indicating that individuals experiencing LBP often have impaired deep trunk muscle coordination and control. Motor control employs principles from motor learning including simplification, segmentation, and task-specific training to reestablish control over the activation, alignment, and movement of the trunk muscles [65]. Accordingly, the first phase of our intervention was to teach/retrain proper activation of deep trunk muscle that were not activating properly. As such, the combination of motor control with ILEX to ensure that trunk muscles are properly activating may be key to lead to observable trunk muscle morphological changes. 

While our findings suggested that a 12-week MC+ILEX intervention (ILEX = two sets of 15–20 repetitions, at a load of 55% of 1 RM, 2 times/week) was effective to improve multifidus and erector spinae muscle size, both interventions had negligeable effect on fatty infiltration. However, there was considerable variation in baseline multifidus and erector spinae values among the participants, ranging from 14% to 58%, making it hard to detect a change. Higher levels of intramuscular fatty infiltration may be more resilient to changes [32] and require higher mechanical stimulus (intensity and frequency) to elicit an exercise-induced reversal of fatty infiltration. Our findings align with prior studies and a recent systematic review that suggested exercise may not reverse paraspinal muscle infiltration, which concluded paraspinal muscle infiltration that is not reversible by means of exercise in chronic LBP. More RCTs with standardized methodologies, larger sample sizes and extended treatment durations are needed to establish a more definitive conclusion [66]. Welch et al. (2015) is the only recent study that reported a significant decrease in lumbar paraspinal muscle fatty infiltration following a 16-week (three sets of 5–8 reps, load at 6–10 RM, 3 times/week), progressive, high-load free-weight resistance training, without an ILEX component [32]. However, that study had important methodological bias, including partial and suboptimal measurements of paraspinal muscle composition and size (both the multifidus and erector spinae were measured together in the same region of interest). While the ideal exercise duration and intensity for reducing intramuscular fatty infiltration remains uncertain [66,67], noticeable chronic changes in the homeostatic myocellular environment are observable after 8 weeks of exercise [68], and could take even longer in untrained individuals [69]. As such, reversing fatty infiltration by means of exercise may require longer interventions with more frequent training sessions that involve greater levels of muscle loading. The need for longer intervention and high-intensity exercise to improve muscle quality and outcomes in LBP has been emphasized in the literature [43,66].

Another factor that may contribute to the discrepancy of literature results is the difference in segmentation and imaging sequence used. To our knowledge, the current study uniquely measured fatty infiltration using IDEAL fat/water images, which is reported to be simpler and more accurate than current methods [70]. Moreover, we took the mean of three slices per spinal level instead of one slice, which allows for a more precise assessment of overall muscle quality [71]. Furthermore, to adequately compare our results to those of other studies, we must consider the region of interest and related segmentation protocol used to acquire paraspinal muscle measurements. Our study included the “epimuscular fat band” when present, which could explain why the fat percentages were higher than in previous studies. Providing more detailed explanations of the imaging and measurement techniques used will help facilitate the comparison of results since various techniques are often used without a detailed description. Our study was primarily composed of females with a percentage that was higher than in previous studies [72]. Larger sample sizes with a more even distribution between sexes are needed to determine if the results can be generalized to both sexes and across various age groups. Further research should continue to evaluate the effect of exercise on muscle quality, considering that increased intramuscular fatty infiltration in the lumbar muscles has been associated with the presence and severity of spinal pain and dysfunction [73,74], the development of recurrent/persistent pain [75], lower physical function [74,76,77], inflammatory dysregulation [78], decreased muscle function [79,80], and poorer surgical outcomes [81,82]. Improving paraspinal muscle quality by means of exercise therapy therefore has the potential to provide a necessary biomarker towards informing and measuring therapeutic success [71,83]. 

In accordance with the increase in CSA, we also observed a significant increase in multifidus thickness at both L4 and L5 levels from baseline and postintervention in the MC+ILEX group, with no change in the GE group. The observed changes surpassed the minimal detectable change of 3.6 mm for resting thickness, implying it was likely a true change rather than one caused by measurement error [33]. These results align with previous studies investigating the impact of motor control exercise on multifidus thickness [33,84]. On the other hand, no significant difference was found in the percent thickness change between rest and contracted states. These findings are consistent with a study conducted by Lariviere et al., which also reported no increase in multifidus activation after an 8-week lumbar stabilization intervention [53]. Limited research has explored the effects of motor control exercise on the percentage change in multifidus thickness within the LBP population [33]. While ultrasound measures of the lumbar multifidus %thickness change is reliable and valid method and commonly used as a proxy to assess muscle function [53], it does not provide any information about the timing of activation. 

With regard to patient-oriented outcomes, we found no between-group difference for any of the outcomes. However, within-group reductions were observed in both the MC+ILEX and GE group. A significant reduction in pain, disability, and psychological health were observed in the MC+ILEX, with a statistically significant decrease in pain across all time points (baseline, 6 weeks, postintervention). There was a 2.4-point improvement in pain scores from baseline to postintervention, which was higher than the minimal clinically important difference (MCID) of 2.0 [85]. The GE group also experienced a statistically significant reduction in pain scores from baseline to postintervention, but these scores did not meet clinical significance. Both groups also experienced a statistically significant decrease in ODI scores across all time points, which was lower than the MDC of 13.5–16.7, although the exact clinical significance is still largely unknown [86,87]. Comparably, Smith et al. 2011 found that lumbar extension training with pelvic stabilization had a positive effect on NPR and ODI scores in the pelvic stabilization group, which was consistent with previous studies [88,89,90]. Furthermore, the SF-12 physical health scores significantly improved by 6.42 points in the MC+ILEX group and 4.69 in the GE group from baseline to postintervention. These results are higher than the MDC of 3.77 and the MCID of 3.29 [91], suggesting that the MC+ILEX intervention led to important physical health benefits in individuals with chronic LBP. Similarly, Steele et al. found that isolated lumbar extension resistance training is effective in reducing pain intensity, improving physical function, and improving quality of life in individuals with chronic LBP [22]. Exercise generally improves mental health by reducing anxiety and depression and improving self-esteem and cognitive function [92]. Interestingly, the SF-12 mental health scores significantly improved from baseline to postintervention in the GE group whereas the MC+ILEX did not. General resistance training includes a variety of exercises that induce repeated muscle contractions against resistance levels beyond those encountered in daily activities [93]. The GE group may have gained strength and endurance through resistance exercises, enabling them to complete everyday tasks that were previously too challenging, resulting in a significant improvement in mental health. Research also suggests that general resistance training offers benefits that extend beyond muscle and tissue growth and include neurobiological alterations relevant to mental health and anxiety-related outcomes [93]. Future research should also evaluate the effect of a combined ILEX and GE intervention on paraspinal muscle health and patient-related outcomes. 

Contrary to our hypothesis, we found no correlation between improvements in muscle health and functional improvements. However, our results revealed correlations between multifidus thickness at L4 (r = −0.31, *p* < 0.05) and L5 (r = −0.37, *p* < 0.05) with changes in SF-12 mental health scores. This suggests that SF-12 mental health scores improve even when multifidus thickness did not. Mental health and physical health are linked. People living with chronic physical conditions are at a higher risk of experiencing a wide range of psychological conditions and vice versa [94,95]. This emphasizes the importance of physical activity and exercise because as physical activity increases, function increases, which positively impacts cognitive function and mental health [96]. We are aware of only one study (*n* = 14) that has reported a significant correlations between functional and MRI-measured outcomes [31]. Specifically, participants who demonstrated improvements in erector spinae muscle health also demonstrated the largest functional improvement in LBP-related disability, depression, and anxiety. Our results, however, did not corroborate these findings. 

While correlation does not imply causation, accumulating evidence suggests that multifidus and erector spinae muscle changes may play a role in driving symptoms in individuals with LBP, rather than solely attributed to immobilization and motion restriction due to pain. Studies by Noonan and Brown and Shi et al. demonstrated that individuals with LBP exhibit a distinct pattern of atrophy and fatty infiltration in these muscles [97,98]. Experimental research, such as that conducted by Shahidi et al. using muscle biopsies from the multifidus muscle in individuals with lumbar spine pathology revealed significant muscle atrophy due to imbalanced degeneration and regeneration processes, increased inflammation, and reduced vascularity [99]. These findings collectively suggest a more complex relationship, where muscle changes likely exacerbate LBP symptoms while also being influenced by pain-induced adaptations. Thus, while immobilization and motion restriction may contribute, multifidus and erector spinae muscle modifications appear to have a distinct impact on LBP symptomatology.

### Limitations

The study presented had multiple strengths, including adopting a randomized controlled design, a high level of adherence to both exercise interventions, and the adoption of reliable and valid outcome measures. Nevertheless, there are also some limitations, including the restricted recruitment of participants caused by COVID-19, which led to limited hours and capacity restrictions at the facility. The participant selection was restricted to those who found out about the opportunity via word of mouth or through connections to Concordia University, rather than from multiple sources. Additionally, due to the nature of the treatments given, the participants were aware of which group they were placed in, making it impossible to blind the parties involved. Additionally, one potential limitation of this study is the difference in professional background and experience of the individuals delivering both interventions. Furthermore, it has been established that males and females have different muscular characteristics. Given that our sample was primarily female, it is more difficult to generalize findings. Due to technical issues with the MR images, it was not possible to assess % fat fraction in five participants. Furthermore, while we originally planned to assess clinical outcomes at 24 weeks postintervention, most participants did not respond to our email request or answered a few weeks afterwards, and thus these data were not included in the analysis. Lastly, the measurements were only taken at the lower lumbar levels (L4/L5 and L5/S1). Our group is currently working on assessing the effect of each intervention at the remaining spinal levels, and their associations with lumbar strength and psychological factors. Pain-related fear and other psychological outcomes were collected and will be reported as part of another related manuscript.

## 5. Conclusions

The findings from this study offer preliminary evidence to support that a MC+ILEX intervention may be effective in improving paraspinal morphology while decreasing pain and disability. However, further research is warranted to confirm these findings and better understand the effects of exercise on overall paraspinal muscle health and its relation to outcomes associated with LBP. The long-term effect of similar exercise interventions should also be investigated in a larger sample size, different spine pathologies, and in the perioperative period.

## Figures and Tables

**Table 1:** Baseline characteristics of the participants within each group.

Group	MC+ILEX *n* = 25	GE *n* = 25	*p*-Value
Age (year) (mean ± SD)	45.16 ± 10.66	37.60 ± 11.60	0.020 ^#^
Sex (male)	5 (33.3%)	10 (66.7%)	0.123 *
Sex (female)	20 (57.1%)	15 (42.9%)	
Height (cm)	169.68 ± 10.93	169.29 ± 7.86	0.887 ^#^
Weight (kg)	75.08 ± 16.39	76.39 ± 19.58	0.805 ^#^
BMI	26.09 ± 5.01	26.40 ± 5.22	0.834 ^#^
LBP Length (months)	73.52 ± 82.81	101.69 ± 105.62	0.299 ^#^
NPR Scores			
Baseline	5.26 ± 1.75	5.19 ± 1.72	0.887 ^#^
6 weeks	3.58 ± 1.78	3.63 ± 1.07	0.890 ^#^
12 weeks	2.80 ± 1.81	3.41 ± 1.62	0.225 ^#^
ODI Scores			
Baseline	29.40 ± 9.85	26.04 ± 10.03	0.238 ^#^
6 weeks	22.96 ± 11.47	21.45 ± 10.05	0.637 ^#^
12 weeks	19.70 ± 10.66	18.27 ± 7.05	0.756 ^#^
SF-12 Scores			
Baseline	87.10 ± 12.88	79.62 ± 27.09	0.218 ^#^
6 weeks	89.39 ± 12.92	85.60 ± 29.43	0.558 ^#^
12 weeks	90.77 ± 24.33	83.53 ± 33.75	0.389 ^#^

Categorical variables are shown as number (%) and numerical data are shown as mean ± SD. * Based on chi square test. ^#^ Based on independent samples *t*-test.

**Table 2:** Adjusted multifidus and erector spinae muscle CSA means in the MC+ILEX and GE groups.

Variables	Measurement Period	MC+ILEX *n* = 25	GE *n* = 25	Main Effect of Group	Interaction Effect between Time and Group
L4/L5 MF CSA (cm^2^)	Baseline	10.00	10.00	*p*-value = 0.009 F = 7.55 df = 1	*p*-value = 0.001 F = 8.33 df = 1.72
	6 weeks (std. error)	10.31 (0.13) *	9.98 (0.14)		
	12 weeks	10.69 (0.15) *	9.90 (0.17)		
	MD (95% CI)	0.69 (0.38 to 1.00) *	−0.10 (−0.43 to 0.24)		
	Main effect of time	*p*-value = <0.001 F = 11.60 df = 2	*p*-value = 0.71 F = 0.35 df = 2		
L4/L5 ES CSA (cm^2^)	Baseline	16.31	16.31	*p*-value = 0.001 F = 12.49 df = 1	*p*-value = 0.002 F = 6.53 df = 2
	6 weeks (std. error)	16.91 (0.21) *	16.11 (0.22)		
	12 weeks	17.48 (0.27) *	16.20 (0.29)		
	MD (95% CI)	1.17 (0.63 to 1.71) *	−0.16 (−0.70 to −0.47)		
	Main effect of time	*p*-value = <0.001 F = 9.94 df = 2	*p*-value = 0.68 F = 0.39 df = 2		
L5/S1 MF CSA (cm^2^)	Baseline	11.88	11.88	*p*-value = <0.001 F = 7.27 df = 1	*p*-value = <0.001 F = 14.47 df = 2
	6 weeks (std. error)	12.10 (0.15)	11.74 (0.17)		
	12 weeks	12.57 (0.15) *	11.43 (0.17) *		
	MD (95% CI)	0.69 (0.39 to 1.00) *	−0.45 (−0.80 to −0.10) *		
	Main effect of time	*p*-value = <0.001 F = 13.08 df = 2	*p*-value = 0.013 F = 4.99 df = 2		
L5/S1 ES CSA (cm^2^)	Baseline	11.28	11.28	*p*-value = <0.001 F = 23.88 df = 1	*p*-value = <0.001 F = 14.54 df = 2
	6 weeks (std. error)	12.29 (0.31) *	10.78 (0.35)		
	12 weeks	13.18 (0.42) *	10.11 (0.47)		
	MD (95% CI)	1.90 (1.06 to 2.73) *	−1.174 (−2.12 to −0.23) *		
	Main effect of time	*p*-value = <0.001 F = 11.04 df = 2	*p*-value = 0.054 F = 3.14 df = 2		

* The mean difference is significant at the 0.05 level.

**Table 3:** Adjusted multifidus and erector spinae muscle % fat fraction means in the MC+ILEX and GE groups.

Variables	Measurement Period	MC+ILEX *n* = 25	GE *n* = 25	Main Effect of Group	Interaction Effect between Time and Group
L4/L5 MF FF (cm^2^)	Baseline	25.91	25.91	*p*-value = 0.891 F = 0.02 df = 1	*p*-value = 0.388 F = 0.96 df = 2
	6 weeks (std. error)	25.89 (0.53)	25.15 (0.52)		
	12 weeks	24.95 (0.67)	25.52 (0.65)		
	MD (95% CI)	−0.96 (−2.31 to 0.39)	−0.39 (−1.71 to 0.92)		
	Main effect of time	*p*-value = 0.37 F = 1.11 df = 2	*p*-value = 0.34 F = 1.11 df= 2		
L4/L5 ES FF (cm^2^)	Baseline	33.48	33.48	*p*-value = 0.96 F = 0.002 df = 1	*p*-value = 0.97 F = 0.03 df = 2
	6 weeks (std. error)	33.21 (0.81)	33.11 (0.79)		
	12 weeks	32.96 (0.59)	33.14 (0.57)		
	MD (95% CI)	−0.52 (−1.70 to 0.67)	−0.34 (−1.50 to 0.82)		
	Main effect of time	*p*-value = 0.68 F = 0.389 df = 2	*p*-value = 0.79 F = 0.235 df = 2		
L5/S1 MF FF (cm^2^)	Baseline	27.92	27.92	*p*-value = 0.37 F = 0.82 df = 1	*p*-value = 0.46 F = 0.80 df = 2
	6 weeks (std. error)	27.80 (0.38)	27.56 (0.39)		
	12 weeks	28.23 (0.53)	27.43 (0.54)		
	MD (95% CI)	0.32 (−0.76 to 1.40)	−0.49 (−1.59 to 0.62)		
	Main effect of time	*p*-value = 0.008 F = 5.62 df = 2	*p*-value = 0.69 F = 0.39 df = 2		
L5/S1 ES FF (cm^2^)	Baseline	41.38	41.38	*p*-value = 0.12 F = 2.56 df = 1	*p*-value = 0.24 F = 1.47 df = 2
	6 weeks (std. error)	39.34 (0.62) *	40.94 (0.63)		
	12 weeks	39.28 (0.79)	40.68 (0.81)		
	MD (95% CI)	−2.09 (−3.70 to −0.49) *	−0.70 (−2.35 to 0.95)		
	Main effect of time	*p*-value = 0.01 F = 5.62 df = 2	*p*-value = 0.69 F = 0.38 df = 2		

* The mean difference is significant at the 0.05 level.

**Table 4:** Adjusted multifidus thickness means at L4 and L5 in the MC+ILEX and GE groups.

Variables	Measurement Period	MC+ILEX *n* = 25	GE *n* = 25	Main Effect of Group	Interaction Effect between Time and Group
L4	Baseline	2.95	2.95	*p*-value = 0.03 F = 4.81 df = 1	*p*-value = <0.001 F = 8.72 df = 2
	6 weeks (std. error)	3.09 (0.03) *	3.00 (0.03)		
	12 weeks	3.17 (0.04) *	2.95 (0.04)		
	MD (95% CI)	0.22 (0.15 to 0.29) *	−0.002 (−0.08 to 0.73)		
	Main effect of time	*p*-value = <0.001 F = 20.91 df = 2	*p*-value = 0.28 F = 1.32 df = 2		
L5	Baseline	2.83	2.83	*p*-value = <0.001 F = 19.04 df = 1	*p*-value = <0.001 F = 11.04 df = 2
	6 weeks (std. error)	2.99 (0.03) *	2.85 (0.03)		
	12 weeks	3.08 (0.04) *	2.87 (0.04)		
	MD (95% CI)	0.25 (0.18 to 0.32) *	0.35 (−0.04 to 0.11)		
	Main effect of time	*p*-value = <0.001 F = 27.06 df = 2	*p*-value = 0.65 F = 0.43 df = 2		

* The mean difference is significant at the 0.05 level; muscle thickness units are expressed in millimeters (mm).

**Table 5:** Adjusted L4 and L5 multifidus for % thickness change (rest to contracted) in the MC+ILEX and GE groups.

Variables	Measurement Period	MC+ILEX *n* = 25	GE *n* = 25	Main Effect of Group	Interaction Effect between Time and Group
L4	Baseline	15.76	15.76	*p*-value = 0.96 F = 0.002 df = 1	*p*-value = 0.79 F = 0.24 df = 2
	6 weeks (std. error)	15.36 (1.31)	14.83 (1.39)		
	12 weeks	15.99 (0.91)	16.65 (0.97)		
	MD (95% CI)	0.22 (−1.60 to 2.05)	0.88 (−1.06 to 2.83)		
	Main effect of time	*p*-value = 0.88 F = 0.13 df = 2	*p*-value = 0.37 F = 1.03 df = 2		
L5	Baseline	11.10	11.10	*p*-value = 0.93 F = 0.007 df = 2	*p*-value = 0.98 F = 0.03 df = 2
	6 weeks (std. error)	10.71 (0.95)	10.43 (1.01)		
	12 weeks	11.06 (1.14)	11.12 (1.21)		
	MD (95% CI)	−0.04 (−2.33 to 2.25)	0.02 (−2.43 to 2.46)		
	Main effect of time	*p*-value = 0.91 F = 0.10 df = 2	*p*-value = 0.77 F= 0.26 df = 2		

**Table 6:** Comparison of pain and disability scores in the MC+ILEX and GE groups.

Variables	Measurement Period	MC + ILEX *n* = 25	GE *n* = 25	Main Effect of Group	Interaction Effect between Time and Group
Pain score (NPR)	Baseline	5.23 ± 0.34	5.20 ± 0.42	*p*-value = 0.52 F = 0.43 df = 1	*p*-value = 0.34 F = 1.10 df = 2
	6 weeks (std. error)	3.58 ± 0.33 *	3.73 ± 0.41 *		
	12 weeks	2.80 ± 0.38 *	3.56 ± 0.46		
	MD (95% CI)	−2.43 (−3.26 to 1.61) *	−1.64 (−2.64 to 0.63)		
	Main effect of time	*p*-value = <0.001 F = 20.69 df = 2	*p*-value = <0.001 F = 8.46 df = 2		
Disability score (ODI)	Baseline	29.54 ± 2.05	27.52 ± 2.19	*p*-value = 0.62 F = 0.25 df = 1	*p*-value = 0.84 F = 0.11 df = 1.49
	6 weeks (std. error)	23.08 ± 2.23 *	22.00 ± 2.38 *		
	12 weeks	19.08 ± 1.95 *	18.19 ± 2.08 *		
	MD (95% CI)	−10.46 (−14.55 to −6.37) *	−9.33 (−13.71 to −4.96) *		
	Main effect of time	*p*-value = <0.001 F = 14.46 df = 2	*p*-value = <0.001 F = 10.40 df = 2		
SF-12 physical (PCS)	Baseline	38.78 ± 1.76	40.75 ± 1.88	*p*-value = 0.29 F = 1.17 df = 1	*p*-value = 0.32 F = 1.14 df = 2
	6 weeks (std. error)	42.26 ± 1.53	46.16 ± 1.64 *		
	12 weeks	45.20 ± 1.51 *	45.44 ± 1.61		
	MD (95% CI)	6.42 (2.67 to 10.18) *	4.70 (0.68 to 8.71) *		
	Main effect of time	*p*-value = 0.004 F = 6.23 df = 2	*p*-value = 0.03 F = 4.04 df = 2		
SF-12 mental (MCS)	Baseline	48.83 ± 2.12	45.67 ± 2.27	*p*-value = 0.71 F = 0.14 df = 1	*p*-value = 0.37 F = 1.02 df = 2
	6 weeks (std. error)	47.03 ± 1.97	46.43 ± 2.11		
	12 weeks	49.34 ± 2.49	49.85 ± 2.67		
	MD (95% CI)	0.52 (−3.07 to 4.10)	4.17 (0.35 to 8.01) *		
	Main effect of time	*p*-value = 0.41 F = 0.90 df = 2	*p*-value = 0.07 F = 2.78 df = 2		

* The mean difference is significant at the 0.05 level.

**Table 7:** Correlations between changes in the muscle morphology and changes in secondary outcomes.

Group	ΔNPR [95% CI]	ΔODI [95% CI]	ΔSF12-M [95% CI]	ΔSF12-P [95% CI]	ΔSF12 [95% CI]
ΔMF CSA L4/L5	0.08 [−0.22 to 0.36]	0.21 [−0.09 to 0.47]	−0.10 [−0.38 to 0.20]	−0.15 [−0.43 to 0.15]	−0.16 [−0.44 to 0.14]
ΔMF CSA L5/S1	−0.13 [−0.42 to 0.18]	−0.09 [−0.38 to 0.22]	0.01 [−0.29 to 0.31]	−0.07 [−0.37 to 0.23]	−0.04 [−0.34 to 0.26]
ΔES CSA L4/L5	0.02 [−0.29 to 0.31]	−0.07 [−0.36 to 0.22]	−0.14 [−0.42 to 0.16]	0.12 [−0.18 to 0.40]	−0.01 [−0.30 to 0.29]
ΔES CSA L5/S1	−0.15 [−0.43 to 0.16]	0.16 [−0.19 to 0.40]	−0.29 [−0.55 to 0.01]	−0.05 [−0.35 to 0.25]	−0.22 [−0.49 to 0.09]
ΔMF FF L4/5	−0.00 [−0.31 to 0.30]	0.22 [−0.10 to 0.49]	0.03 [−0.28 to 0.33]	−0.10 [−0.39 to 0.22]	−0.05 [−0.35 to 0.27]
ΔMF FF L5/S1	−0.16 [−0.45 to 0.17]	−0.32 [−0.57 to 0.00]	0.06 [−0.26 to 0.37]	0.18 [−0.15 to 0.47]	0.15 [−0.17 to 0.45]
ΔES FF L4/L5	−0.02 [−0.32 to 0.29]	0.21 [−0.11 to 0.49]	−0.08 [−0.38 to 0.24]	−0.13 [−0.42 to 0.18]	−0.14 [−0.43 to 0.18]
ΔES FF L5/S1	0.12 [−0.20 to 0.42]	0.14 [−0.18 to 0.44]	0.03 [−0.29 to 0.34]	−0.07 [−0.38 to 0.25]	−0.03 [−0.34 to 0.29]
ΔMF Thickness L4	−0.05 [−0.34 to 0.25]	−0.12 [−0.40 to 0.18]	−0.31 * [−0.55 to −0.01]	0.06 [−0.24 to 0.34]	−0.16 [−0.43 to 0.14]
ΔMF Thickness L5	0.02 [−0.27 to 0.31]	−0.08 [−0.37 to 0.22]	−0.37 * [−0.60 to −0.09]	0.16 [−0.14 to 0.43]	−0.13 [−0.41 to 0.17]

* Indicates *p* < 0.05.

## Data Availability

All extracted data are available upon request.

## Appendix A

**Table A1:** Two-day exercise program performed by the GE group [1].

Day 1	Day 2
Hip extension (multi-hip machine) *	Goblet squat
Prone leg curl *	Step up
Lat pull-down *	Leg extension *
Seated row *	Peck deck *
Hip abduction *	Lying side hip raises
Hip adduction	Abdominal curl

* Exercise executed on a resistance machine.

**Table A2:** Sample cues and positions for early transverse abdominis and multifidus activation [1].

Multifidus Activation	
Positions	Prone or on hands and knees (depending on individuals preference)
	Place fingers on either side of spinous process; assess various spinal levels from T1/T2 to L5/S1
Cues	Attempt to swell muscle up towards fingers
	Mentally visualize tilting pelvis without physically executing the movement
	Visualize contracting a cable that runs from your pelvis through your spine
Ideal	Symmetrical contraction
response	Absence of activation of the global muscles
	Normal breathing
	Able to hold 10 × 10 s
Transverse Abdominis Activation	
Positions	Initial position or crook-lying
	Find neutral pelvis
	Position fingers slightly towards the midline and below the anterior superior iliac spine (ASIS)
Cues	Attempt to draw your navel downwards to the table
	Attempt to move your fingers together (medially)
Ideal	Gradually increase tension; exerting a 10–15% level of effort
response	Symmetrical contraction
	Absence of activation of the global muscles
	Normal breathing
	Able to hold 10 × 10 s
